# Trends in Utilization of Electronic Consultations Associated With Patient Payer and Language Among US Academic Medical Centers During the COVID-19 Pandemic

**DOI:** 10.1001/jamanetworkopen.2022.24628

**Published:** 2022-07-29

**Authors:** Anita Arora, Renee Fekieta, Zakia Nouri, Danielle Carder, Megan M. Colgan, Anne Fuhlbrigge, Sara L. Jackson, Samuel Collins, Nathaniel Gleason, Julia Chen

**Affiliations:** 1 Yale School of Medicine, New Haven, Connecticut; 2 Association of American Medical Colleges, Washington, District of Columbia; 3 Dartmouth-Hitchcock Medical Center, Lebanon, New Hampshire; 4 University of Colorado School of Medicine, Aurora; 5 University of Washington School of Medicine, Seattle; 6 University of Virginia, Charlottesville; 7 University of California, San Francisco; 8 University of Michigan, Ann Arbor

## Abstract

**Importance:**

Electronic consultations (eConsultations) are increasingly used to obtain specialist guidance, avoiding unnecessary face-to-face patient visits for certain clinical questions. During the COVID-19 pandemic, when in-person care was limited, eConsultations may have helped clinicians obtain specialist input to guide patient care.

**Objective:**

To understand how the use of eConsultations changed during the COVID-19 pandemic and whether trends in eConsultation utilization differed based on patient’s payer and primary language.

**Design, Setting, and Participants:**

This retrospective cohort study was conducted at 6 academic medical centers in the United States, all participating in the Association of American Colleges Coordinating Optimal Referral Experiences program. Participants included adult patients who had an outpatient visit, referral, or eConsultation during the study period. Data were analyzed from June 4, 2019, to July 28, 2020.

**Main Outcomes and Measures:**

The primary outcome was the eConsultation proportion of specialty contact, defined as the number of completed eConsultations divided by the sum of the number of completed eConsultations and specialty referrals, expressed as a percentage. eConsultation percentages of specialty contact were further stratified by payer type and language. Payers included commercial, Medicare, Medicaid, self-pay or uninsured, and other. Primary language included English and non-English languages.

**Results:**

A total of 14 545 completed eConsultations and 189 776 referrals were included. More eConsultations were completed for English-speaking patients (11 363 eConsultations [95.0%]) than non-English–speaking patients (597 eConsultations [5.0%]). Patients with commercial insurance represented the highest number of completed eConsultations (8848 eConsultations [60.8%]) followed by Medicare (3891 eConsultations [26.8%]), Medicaid (930 eConsultations [6.4%]), other insurance (745 eConsultations [5.1%]), and self-pay or no insurance (131 eConsultations [0.9%]). At the start of the pandemic, across all academic medical centers, the percentage of specialty contact conducted via eConsultation significantly increased by 6.21% (95% CI, 4.97%-7.44%; *P* < .001). When stratified by payer and language, the percentage of specialty contact conducted via eConsultation significantly increased at the beginning of the pandemic for both English-speaking patients (change, 6.09% (95% CI, 4.82% to 7.37%; *P* < .001) and non-English–speaking patients (change, 8.48% [95% CI, 5.79% to 11.16%]; *P* < .001) and for all payers, except self-pay and uninsured patients (change, −0.21% [95% CI, [−1.35% to 0.92%]; *P* = .70).

**Conclusions and Relevance:**

This retrospective cohort study found that eConsultations provided an accessible mechanism for clinicians to receive specialist input when in-person care was limited.

## Key Points

**Question:**

How did utilization of electronic consultations (eConsultations) change during the COVID-19 pandemic for US academic medical centers with mature eConsultation programs?

**Findings:**

In this cohort study including 14 545 completed eConsultations, eConsultations as a proportion of all specialty consultations significantly increased in the first week of the pandemic. This held true for both English- and non-English–speaking patients and across all payer types, except for self-pay and uninsured patients.

**Meaning:**

These findings suggest that eConsultations provided an accessible mechanism for clinicians to receive specialist input when in-person care was limited.

## Teaser

This cohort study examines how the use of electronic consultations has changed during the COVID-19 pandemic in the US and whether trends in use differed based on patient’s payer and primary language.

## Introduction

Electronic consultations, or *eConsultations*, are increasingly used by primary care clinicians to obtain specialty input.^1^ eConsultations are asynchronous clinical exchanges between health care clinicians, facilitated within the electronic health record (EHR) or a web-based platform. Prior to the COVID-19 pandemic, eConsultations had been shown to reduce unnecessary face-to-face referrals to specialty clinics and provide expanded and more timely access to specialists.^2,3^ However, there is limited information about how eConsultations were used during the pandemic across multiple health systems.

The early months of the COVID-19 pandemic demonstrated a need for patient care that limited in-person clinical encounters, thereby reducing potential exposure to COVID-19 and preserving personal protective equipment. In addition, the pandemic increased pressure to deliver accessible, high-quality health care while improving efficiency in the face of limited resources. Furthermore, government payers and private insurers promoted the use of telemedicine through relaxed requirements, allowing health care clinicians increased flexibility to provide remote patient care.

A 2020 study by Lee and Nambudiri^4^ highlighted the important role that eConsultations could serve in providing safe and equitable specialist input in the context of the COVID-19 pandemic; however, the role eConsultations play in the pandemic response has not been broadly reported. A study by Phadke et al^5^ demonstrated that after Massachusetts declared a state of emergency in early March 2020, eConsultations as a proportion of total consultation volume (both eConsultations and referrals for face-to-face visits) increased by 5%. However, this report was from a single academic medical center (AMC). Further study is needed to grasp the broader effect of the pandemic on eConsultation utilization, to develop an understanding of the role of eConsultations during a public health emergency, and to consider potential benefits of eConsultations as virtual augmentation of patient care continues to meet health care needs.

In this study, we incorporated eConsultation, referral, and visit data from 6 AMCs from June 2019 through July 2020 to understand how eConsultation utilization changed during the pandemic. Data were stratified by patient payer and by primary language to explore patterns of eConsultation use and how they differed across various patient populations. We hypothesized that during the COVID-19 pandemic, eConsultation utilization as a percentage of all outreach to specialty care would increase from prepandemic levels and that trends in eConsultation utilization during this period may differ based on patient payer and primary language.

## Methods

### Study Design and Data Sources

We performed a retrospective cohort study of patients cared for at 6 AMCs in the United States (Dartmouth-Hitchcock Medical Center; University of California, San Francisco; University of Colorado; University of Michigan; University of Washington; and Yale University). All 6 AMCs participate in the Association of American Medical Colleges’ (AAMC) Coordinating Optimal Referral Experiences (CORE) program. Clinicians at all 6 AMCs use the EHR to place and respond to eConsultations. The study protocol was granted exemption from review and informed consent by the institutional review boards at each of the AMCs because it met the criteria for quality improvement designation. This study is reported following the Strengthening the Reporting of Observational Studies in Epidemiology (STROBE) reporting guideline for cohort studies.

Data from the 6 AMCs incorporated 3 components: completed eConsultations, referrals to specialists, and primary care visits differentiated by visit type (ie, in-person, video, or phone). Patient data were extracted from the EHR from June 4, 2019, through July 28, 2020, and then summarized by weekly intervals for each AMC. The patient population included adults aged 18 years as of January 1, 2019. Patients’ payer type and primary language were also included for all completed eConsultations and specialty referrals. We compiled a list of the most common and frequently consulted medical and surgical specialties across the 6 AMC sites and included those that had started providing eConsultations before May 20, 2019 (eTable 1 in the Supplement). eConsultations that were completed by the specialists and did not require an in-person visit were included in the analysis.

Referrals to specialists included internal referrals as well as external referrals that were outside the AMC’s health system. Referrals for directly scheduled procedures, such as colonoscopies, pulmonary function tests, and cardiac stress tests, were excluded because these procedures and tests could not be replaced by an eConsultation. Primary care visits were defined as completed evaluation and management encounters and included both in-person, video, and phone visits. We included primary care visits with clinicians who are able to bill for evaluation and management codes and send eConsultations (eg, physicians and advanced practice clinicians).

### Descriptive Analysis

Data were categorized into a period before the pandemic (June 4, 2019, through March 3, 2020) and during the initial phase of the pandemic (March 4, 2020, through July 28, 2020). Together, these 2 periods made up 60 weeks of data included in the study. For each AMC, we calculated eConsultation percentage of specialty contact in the prepandemic and pandemic periods.

For each period, we also calculated mean monthly volumes of telehealth visits in primary care for each institution and overall, and mean monthly volumes of eConsultations and specialty referrals. We also calculated eConsultations per 1000 primary care visits, and specialty referrals per 1000 primary care visits, before and during the pandemic, to understand how utilization of eConsultations and specialty referrals changed relative to primary care contact. We calculated the percentage of primary care visits that were conducted via phone or video (phone and video visits as a proportion of total primary care visits) before and during the pandemic to understand the telehealth transition during the pandemic and to contextualize changes in the eConsultation percentage of specialty contact.

### Statistical Analysis

Our primary outcome was weekly eConsultation percentage of specialty contact. We calculated this outcome by aggregating across the AMCs weekly counts of completed eConsultations and referrals to calculate an overall weekly eConsultation percentage of specialty contact (eConsultations / (eConsultations + referrals) × 100). We ran 3 ordinal least square models using interrupted time-series analysis on the weekly eConsultation percentage of specialty contact to assess the association of the pandemic with eConsultation percentage.^6,7^ More specifically, in the first model, we assessed whether the eConsultation percentage of specialty contact had increased during and after the first week of the pandemic (week 40) compared with the prepandemic period, calculated as

*Y_t_* = β_0_ + β_1_ × Time*_t_* + β_2_ × *X_t_* + β_3_ × *X_t_* × Time*_t_* + *ε_t_*

where *Y_t_* is the weekly aggregate eConsultation percentage of specialty contact, and *Time_t_* is a continuous variable that indicates the number of weeks passed since the start of the study corresponding to June 4, 2019, through March 3, 2020, and during the first phase of the pandemic, corresponding to March 4, 2020, through July 28, 2020. *X_t_* is a dummy variable indicating the period prior (coded as 0) and during and after the start of the pandemic (coded as 1) with the 40th week being the interruption week. Finally, *X_t_Time_t_* is a continuous variable which measures the immediate effect of the pandemic on the eConsultation percentage of specialty.

In the second and third models, we stratified by language (English and non-English) and payer type, respectively. In each model, we analyzed the eConsultation percentage of specialty contact at the beginning of the study period, the weekly change in eConsultation percentage of specialty contact before the pandemic (slope), the immediate outcome associated with the pandemic in the eConsultation percentage of specialty contact, the weekly change in eConsultation percentage after week 40 (slope), the change in the slope between prepandemic and pandemic periods, and the counterfactual slope. Analyses were completed using Stata software, version 14.2 (StataCorp). *P* values were 1-sided, and statistical significance was set at *P* = .05. Data were analyzed from June 4, 2019 to July 28, 2020.

## Results

### Descriptive Results

A total of 14 545 completed eConsultations and 189 776 referrals were included. Most completed eConsultations and primary care visits were for English-speaking patients (11 363 eConsultations [95.0%]). This is in comparison to 597 eConsultations (5.0%) for non-English–speaking patients. Most eConsultations were for patients with commercial insurance (8848 eConsultations [60.8%]). Medicare represented the next highest insurance category (3891 eConsultations [26.8%]), followed by Medicaid (930 eConsultations [6.4%]), other (eg, Worker’s Compensation, Tricare for military members; 45 eConsultations [5.1%]), and self-pay or uninsured (131 eConsultations [0.9%]) (Table 1; eTable 2 in the Supplement). Mean monthly specialty in-person referral and eConsultation volumes during the pandemic declined compared with before the pandemic for all AMCs except 1 (eTable 3 in the Supplement). However, across all 6 AMCs, eConsultation percentage of specialty contact increased during the pandemic compared with before the pandemic because eConsultations declined less than referrals (eTable 4 and eFigure in the Supplement). Furthermore, when aggregated across all AMCs, eConsultations increased from 8.4 per 1000 primary care visits prepandemic to 9.1 per 1000 primary care visits during the pandemic compared with referrals, which decreased from 120.5 per 1000 primary care visits prepandemic to 95.0 per 1000 primary care visits during the pandemic (eTable 5 in the Supplement).

**Table 1.** Patient Demographics Stratified by Payer and Primary Language

Characteristic	No. (%)	
	Completed electronic consultations	Total primary care visits
Payer type		
Medicare	3891 (26.8)	463 889 (27.5)
Medicaid	930 (6.4)	167 837 (9.9)
Commercial	8848 (60.8)	990 862 (58.7)
Other	745 (5.1)	50 022 (3.0)
Self-pay and uninsured	131 (0.9)	15 209 (0.9)
Primary language^a^		
English	11 363 (95.0)	1 470 455 (94.2)
Non-English	597 (5.0)	89 754 (5.8)

^a^ Patient primary language for patients at 1 included center was unavailable. A total of 1866 primary care visits with unknown language type were excluded from the percentage of total primary care visits by language.

Monthly telehealth (phone and video) visits in primary care increased during the pandemic. Across all sites, the proportion of telehealth visits increased from 4252 of 1 154 159 prepandemic visits (0.4%) to 248 702 of 533 660 visits (46.6%) during the pandemic (eTable 6 in the Supplement).

### Interrupted Time-Series Analysis of eConsultation Percentage of Specialty Contact

**Figure 1.**
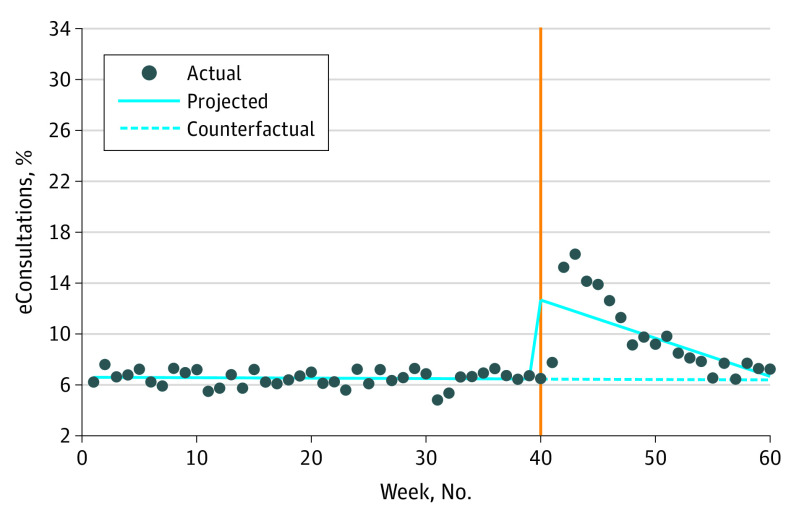
Weekly Electronic Consultations (eConsultations) as a Percentage of Specialty Contact Orange line indicates beginning of COVID-19 pandemic.

At the beginning of our study period, the eConsultation percentage of specialty contact was fairly stable (change from week 0-40, −0.003% [95% CI, −0.013% to 0.006%]; *P* = .47). During the first week of the pandemic (week 40), eConsultation percentage of specialty contact significantly increased by 6.21% (95% CI, 4.96% to 7.44%; *P* < .001). This was followed by a decrease by 0.29% every week (95% CI, −0.39% to −0.20%; *P* < .001). This slope or weekly decrease in eConsultation percentage of specialty contact was significantly lower than during the prepandemic period (change, −0.29%; [95% CI, −0.38 to −0.20]; *P* < .001) (Table 2 and Figure 1).

**Table 2.** Ordinary Least Squares for the Weekly Electronic Consultations as a Percentage of Specialty Contact During the Study Period by Overall, Language, and Payer Type

Outcome variable	% (95% CI)				Counterfactual slope, %
	Prepandemic slope	First wk of COVID-19, change in intercept	During and after the second wk of COVID-19 pandemic, slope	Change in slope from prepandemic to after second wk of pandemic	
Overall	−0.003 (−0.013 to 0.006)	6.21 (4.96 to 7.44)^a^	−0.29 (−0.39 to −0.20)^a^	−0.29 (−0.38 to −.20)^a^	−0.003
Patient Language					
Non-English	0.035 (−0.012 to 0.084)	8.48 (5.79 to 11.16)^a^	−0.54 (−0.73 to −0.34)^a^	−0.57 (−0.77 to −0.37)^a^	0.035
English	−0.009 (−0.022 to 0.003)	6.09 (4.82 to 7.37)^a^	−0.28 (−0.37 to −0.18)^a^	−0.27 (−0.36 to −0.17)^a^	−0.009
Patient payer type					
Commercial	−0.009 (−0.022 to 0.004)	6.93 (5.54 to 8.32)^a^	−0.31 (−0.41 to −0.21)^a^	−0.31 (−0.41 to −0.20)^a^	−0.009
Medicaid	0.009 (−0.016 to 0.034)	2.94 (1.70 to 4.18)^a^	−0.15 (−0.25 to −0.05)^a^	−0.16 (−0.27 to −0.05)^a^	0.009
Medicare	0.007 (−0.002 to 0.017)	5.87 (4.54 to 7.20)^a^	−0.33 (−0.43 to −0.23)^a^	−0.34 (−0.43 to −0.24)^a^	0.007
Other	−0.02 (−0.08 to 0.04)	8.94 (6.44 to 11.43)^a^	−0.28 (−0.48 to −0.08)^a^	−0.26 (−0.48 to −0.04)^b^	−0.02
Uninsured (self-pay)	−0.06 (−0.09 to −0.03)^a^	−0.21 (−1.35 to 0.92)	0.02 (−0.06 to 0.10)	0.08 (−.006 to 0.18)	−0.06

^a^ *P* < .001.

^b^ *P* < .05.

**Figure 2.**
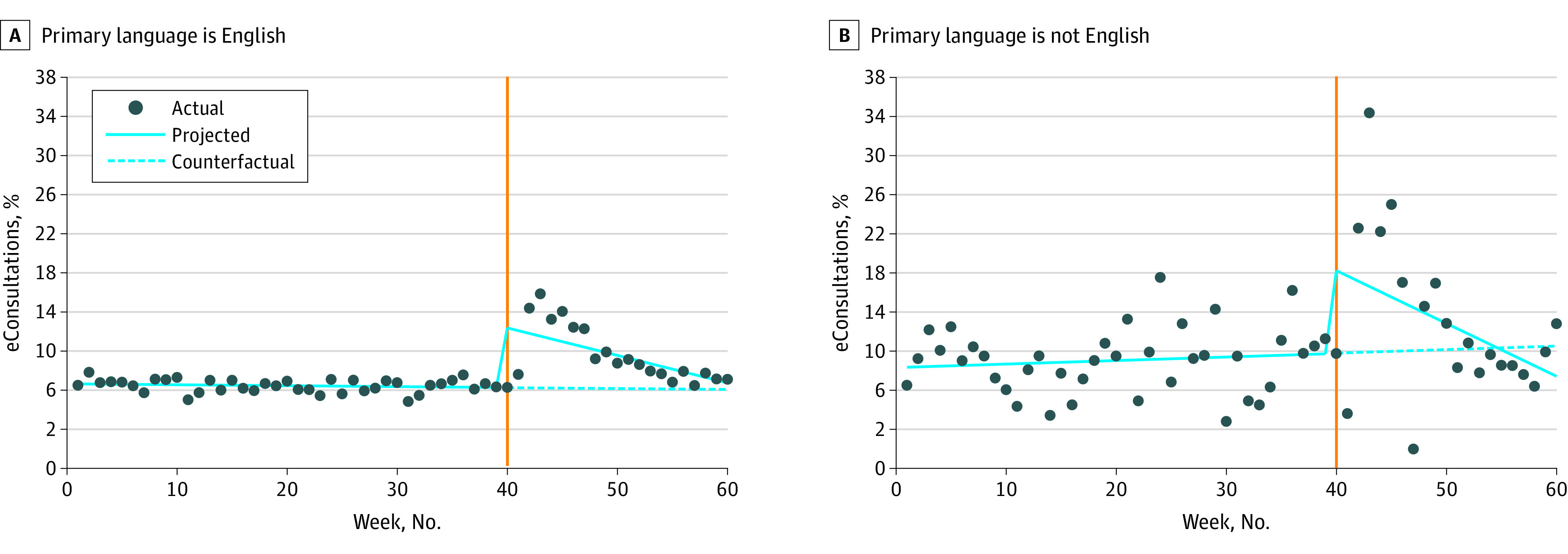
Electronic Consultations (eConsultations) as a Percentage of Specialty Contact by Patient Primary Language Orange line indicates beginning of COVID-19 pandemic.

When stratified by language, we found similar results, with the prepandemic eConsultation percentage of specialty contact fairly stable until the first week of the pandemic, when eConsultation percentage of specialty contact significantly increased for both non-English-speaking patients (change, 8.48% [95% CI, 5.79% to 11.16%]; *P* < .001) and English-speaking patients (change, 6.09% (95% CI, 4.82% to 7.37%; *P* < .001). This was followed by a weekly decrease by −0.54% (95% CI, −0.73% to −0.34%]; *P* < .001) for non-English–speaking patients and by −0.28% (95% CI, −0.37% to −0.18%; *P* < .001) for English-speaking patients. This slope or weekly decrease in eConsultation percentage of specialty contact was significantly lower than during the prepandemic period for both non-English–speaking patients (change, −0.57% [95% CI, −0.77% to −0.37%]; *P* < .001) and English-speaking patients (change, −0.27% [95% CI, −0.36% to −0.17%]; *P* < .001) (Table 2 and Figure 2).

**Figure 3.**
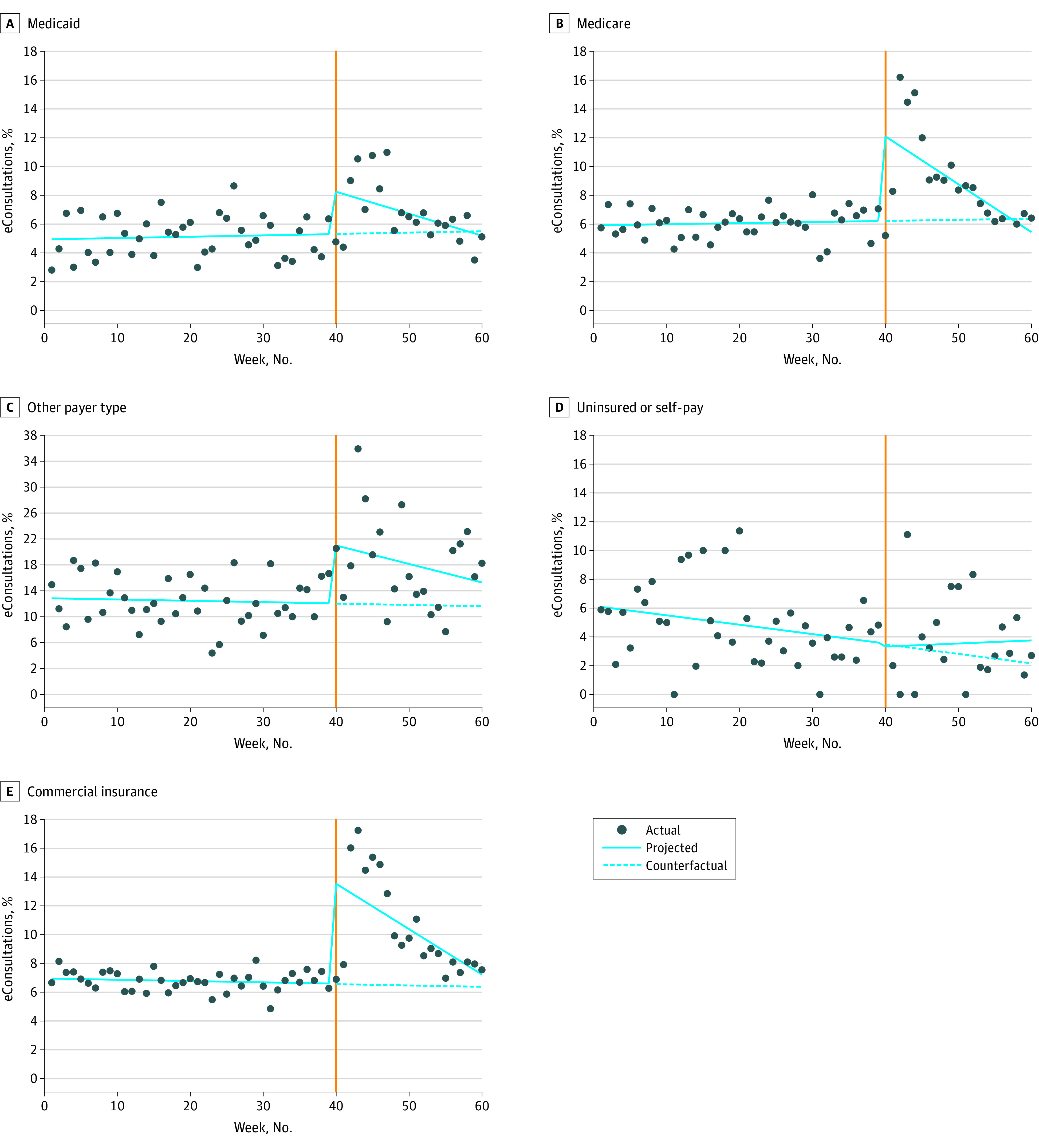
Electronic Consultations (eConsultations) as a Percentage of Specialty Contact by Payer Orange line indicates beginning of COVID-19 pandemic.

Finally, when stratified by payer, we found that the eConsultation percentage of specialty contact was again fairly steady until the first week of the pandemic, when there was a significant increase across all the payer categories, except for the uninsured and self-pay category, for which there was no significant difference (change, −0.21% [95% CI, [−1.35% to 0.92%]; *P* = .70) (Table 2 and Figure 3). During and after the second week of the pandemic, the eConsultation percentage of specialty contact decreased every week by −0.31% (95% CI, −0.41% to −0.21%; *P* < .001) for commercial insurance, −0.15% (95% CI, −0.25% to −0.05%; *P* < .001) for Medicaid, −0.33% (95% CI, −0.43% to −0.23%; *P* < .001) for Medicare, and −0.28% (95% CI, −0.48% to −0.08%]; *P* = .005) for other insurance. The change in eConsultation percentage of specialty contact in the uninsured and self-pay category was not statistically significant. The slope or weekly decrease in eConsultation percentage of specialty contact was significantly lower than it was during the prepandemic period across all the payer categories excluding the uninsured and self-pay group (Figure 3 and Table 2). Full model results are included in eTable 7 in the Supplement.

## Discussion

In this multicenter cohort study of eConsultation use before and after the onset of the COVID-19 pandemic, the eConsultation percentage of specialty contact significantly increased in the first week of the pandemic followed by a steady decline for the rest of our study period. Early in the pandemic, primary care visits, referrals, and eConsultation volumes all decreased; yet, the eConsultation percentage of specialty contact increased, because eConsultation utilization decreased at a lesser rate than referrals overall. After the first week of the pandemic, eConsultation percentage of specialty contact trended down toward the prepandemic level, suggesting that the higher eConsultation percentage of specialty contact in the first few weeks reflected the time it took for health systems to build up telehealth capability and accept more referrals for virtual care. eConsultations played a critical role in access to specialist input during this transition to telehealth.

We observed an increase in eConsultation percentage of specialty contact at the start of the pandemic for both English- and non-English–speaking patients and across all payer groups, except for self-pay and uninsured patients. Concurrently, primary care telehealth visits, including phone and video visits, increased from 0.4% to 46.6% of total visits. While the pandemic was associated with decreased visit volumes overall and a marked increase in the use of telehealth in primary care, centers with established eConsultation programs were able to leverage eConsultations to increase the proportion of virtual specialty care during the pandemic.

This study expands on prior a single-institution study by Phadke et al^5^ that reported an increase in eConsultation utilization using a time frame up to 21 days after the beginning of the pandemic. Prior studies have shown lower use of video-based telehealth during the pandemic by populations at increased risk, including older individuals and non-English speakers.^8,9^ However, our data showed that eConsultation percentage of specialty contact increased regardless of a patient’s primary language and for most payers, except for those who were uninsured or self-pay. In contrast to telehealth visits, eConsultations do not rely on a patient’s access to the internet, ability to speak English, and comfort with the technology needed for a video visit. Therefore, eConsultations may serve as a mechanism to overcome barriers faced by some patients in accessing specialty care during a pandemic.

We found that for patients in the uninsured and self-pay category, eConsultation percentage of specialty contact decreased during the pandemic compared with before the pandemic. It is possible that our sample size was too small to detect differences for this group. However, this result could also indicate that clinicians were less willing to send an eConsultation for a patient who did not have the financial means to follow recommendations during the pandemic, which may include testing or treatment.

While eConsultation proportion of specialty contact increased during the COVID-19 pandemic, we also observed an increase in telehealth visits. Traditionally, a principal benefit of eConsultations is the reduction in barriers to care, including need for transportation, childcare, or excuse from work. Telehealth visits have similar conveniences but still take up time on the specialist’s clinic schedule and require a relationship to be formed between the patient and the specialist. In contrast, eConsultations help to avoid unnecessary specialist visits and allow primary care clinicians to continue driving patient care with specialist input. Future research assessing preferences of patients, primary care clinicians, and specialists, as well as clinical outcomes, is needed to better understand the complementary roles of eConsultations and telehealth platforms in specialty care.

Strengths of this study include data from 6 AMCs that had similarly structured eConsultation programs, including 15 specialties and a total of more than 14 000 eConsultations during the study period. There was diversity in the AMCs themselves as well as the populations they serve—from urban to rural settings, varying primary care population sizes, and different ratios of primary care to specialty care clinicians within each institution. Despite the variations across these centers, this analysis showed similar trends in eConsultation use and uptake. In addition, we include data addressing equity and report 14 months of data, which helps to address seasonal variation in eConsultation utilization.

### Limitations

There are several limitations of this study. First, we are not able to comment on qualitative changes in the content of eConsultations during the pandemic; for example, we cannot comment on whether questions were more complex owing to lack of access to in-person specialty visits. Second, although the pandemic affected different parts of the United States at variable times, we chose to define the start of the pandemic on March 4, 2020, for ease of analysis and discussion. In addition, these results may not be generalizable to all health systems, especially those that do not belong to the AAMC’s Project CORE program or do not have an established eConsultation program. Also billing practices and reimbursement for eConsultations vary across states and changed for many institutions during the pandemic. Our study did not assess the potential association of reimbursement with eConsultation utilization, but this would be an important focus for future work. In addition, primary language and insurance status may have been correlated, but we were unable to evaluate this owing to limitations in the data structure. Furthermore, we did not link eConsultation and referral utilization to the type of primary care visit, so could not determine if eConsultation proportion was higher for telehealth or in-person visits.

## Conclusions

In this retrospective cohort study, we found that eConsultation percentage of specialty contact increased in the first week of the pandemic and trended downward again toward prepandemic levels over the coming weeks. In the early weeks of the pandemic, eConsultations helped to keep clinicians and patients safe by minimizing face-to-face care and played a critical role in providing specialist input in a timely manner to meet the needs of patients and their primary care clinicians.

## Notes

Supplement. **eTable 1.** Specialties Included From 6 Academic Medical Centers **eTable 2.** Completed eConsultations, Completed eConsultations and Referrals, and Percentage eConsultation Specialty Contact Before and During the Pandemic, Stratified by Patient Payer and Primary Language **eTable 3.** Mean Monthly Volumes Before and During the Pandemic **eTable 4.** Completed eConsultations, Completed eConsultations and Referrals, and Percentage eConsultation Specialty Contact, Before and During the Pandemic **eFigure.** eConsultations and Referrals, and Primary Care Visits Over Time **eTable 5.** Number of eConsultations and Referrals per 1000 Primary Care Visits Before and During the Pandemic **eTable 6.** Percentage of Telehealth Visits in Primary Care **eTable 7.** Full Interrupted-Time Series Models
