# Supplementary material for: Trends in Utilization of Electronic Consultations Associated With Patient Payer and Language Among US Academic Medical Centers During the COVID-19 Pandemic
Source: JAMA Netw Open. 2022 Jul 29;5(7):e2224628. doi: 10.1001/jamanetworkopen.2022.24628 (PMC9338406; doi:10.1001/jamanetworkopen.2022.24628)
Supplement: Supplement. — eTable 1. Specialties Included From 6 Academic Medical Centers eTable 2. Completed eConsultations, Completed eConsultations and Referrals, and Percentage eConsultation Specialty Contact Before and During the Pandemic, Stratified by Patient Payer and Primary Language eTable 3. Mean Monthly Volumes Before and During the Pandemic eTable 4. Completed eConsultations, Completed eConsultations and Referrals, and Percentage eConsultation Specialty Contact, Before and During the Pandemic eFigure. eConsultations and Referrals, and Primary Care Visits Over Time eTable 5. Number of eConsultations and Referrals per 1000 Primary Care Visits Before and During the Pandemic eTable 6. Percentage of Telehealth Visits in Primary Care eTable 7. Full Interrupted-Time Series Models [file jamanetwopen-e2224628-s001.pdf]

Supplemental Online Content

Arora A, Fekieta R, Nouri Z, et al. Trends in utilization of electronic consultations associated with patient payer and language among US academic medical centers during the COVID-19 pandemic. *JAMA Netw Open*. 2022;5(7):e2224628. doi:10.1001/jamanetworkopen.2022.24628

eTable 1. Specialties Included From 6 Academic Medical Centers

eTable 2. Completed eConsultations, Completed eConsultations and Referrals, and Percentage eConsultation Specialty Contact Before and During the Pandemic, Stratified by Patient Payer and Primary Language

eTable 3. Mean Monthly Volumes Before and During the Pandemic

eTable 4. Completed eConsultations, Completed eConsultations and Referrals, and Percentage eConsultation Specialty Contact, Before and During the Pandemic

eFigure. eConsultations and Referrals, and Primary Care Visits Over Time

eTable 5. Number of eConsultations and Referrals per 1000 Primary Care Visits Before and During the Pandemic

eTable 6. Percentage of Telehealth Visits in Primary Care

eTable 7. Full Interrupted Time-Series Models

This supplemental material has been provided by the authors to give readers additional information about their work.

© 2022 Arora A et al. *JAMA Network Open.*

eTable 1. Specialties Included From 6 Academic Medical Centers

	A	B	C	D	E	F
Allergy & Immunology		X	X	X	X	
Cardiology	X	X	X	X	X	X
Dermatology			X	X	X	
Endocrinology	X	X	X	X	X	X
ENT	X		X	X		
GI	X	X	X	X	X	
Hematology		X	X	X	X	
Hepatology	X	X	X	X		
Infectious Disease	X	X	X	X	X	
Nephrology	X	X	X	X	X	
Neurology	X	X	X	X	X	X
Orthopedics		X		X		
Pulmonology		X	X	X	X	
Rheumatology	X	X	X	X	X	
Urology	X	X	X	X	X	

Note. Medical and surgical specialties providing eConsults before May 20, 2019 were included.

© 2022 Arora A et al. *JAMA Network Open.*

eTable 2. Completed eConsultations, Completed eConsultations and Referrals, and Percentage eConsultation Specialty Contact Before and During the Pandemic, Stratified by Patient Payer and Primary Language

		**Pre-pandemic (6/4/2019 – 3/3/2020)**			**During pandemic (3/4/2020 – 7/28/2020)**		
		Numerator (eConsults)	Denominator (eConsults +referrals)	% eConsult specialty contact	Numerator (eConsults)	Denominator (eConsults +referrals)	% eConsult specialty contact
Payer	Medicare	2,668	44,270	6.0%	1,223	15,805	7.7%
	Medicaid	616	12,197	5.1%	314	5,034	6.2%
	Commercial	5,859	86,398	6.8%	2,989	32,007	9.3%
	Other	465	3,738	12.4%	280	1,624	17.2%
	Self-pay and uninsured	96	2,070	4.6%	35	1,032	3.4%
Primary Language	English	7,539	116,765	6.5%	3,824	44,371	8.6%
	Non-English	425	4,712	9.0%	172	1,580	10.9%

Notes. AMC A had 146 referrals with unknown payer type that were excluded from the payer analysis. Patient primary language for AMC D was unavailable.

© 2022 Arora A et al. *JAMA Network Open.*

eTable 3. Mean Monthly Volumes Before and During the Pandemic

Pre-Pandemic	Mean Monthly eConsults	Mean Monthly Referrals	Mean Monthly Primary Care Telehealth Visits	Mean Monthly Total Primary Care Visits
A	158	1,743	108	19,486
B	203	4,336	0	28,383
C	238	3,425	8	23,583
D	193	2,840	118	8,810
E	257	2,839	225	39,302
F	29	270	13	8,676
Overall	1,078	15,453	472	128,240

During Pandemic	Mean Monthly eConsults	Mean Monthly Referrals	Mean Monthly Primary Care Telehealth Visits	Mean Monthly Total Primary Care Visits
A	164	1,349	6,778	14,501
B	174	3,083	5,822	20,945
C	217	2,024	9,104	16,908
D	169	1,749	6,828	9,277
E	220	1,787	16,810	38,844
F	26	149	4,398	6,257
Overall	968	10,140	49,740	106,732

Note. During the pandemic, there was a 33% decrease in mean monthly eConsults and referrals combined accompanied by a 17% decrease in mean monthly total primary care visits.

© 2022 Arora A et al. *JAMA Network Open.*

eTable 4. Completed eConsultations, Completed eConsultations and Referrals, and Percentage eConsultation Specialty Contact, Before and During the Pandemic

	**Pre-pandemic (6/4/2019 – 3/3/2020)**			**During pandemic (3/4/2020 – 7/28/2020)**		
Academic Medical Center	Numerator (completed eConsults)	Denominator (completed eConsults +referrals)	% eConsult specialty contact	Numerator (completed eConsults)	Denominator (completed eConsults +referrals)	% eConsult specialty contact
A	1,421	17,106	8.3%	818	7,563	10.8%
B	1,828	40,849	4.5%	868	16,281	5.3%
C	2,139	32,965	6.5%	1,084	11,203	9.7%
D	1,740	27,304	6.4%	845	9,589	8.8%
E	2,317	27,871	8.3%	1,098	10,033	10.9%
F	259	2,686	9.6%	128	871	14.7%

© 2022 Arora A et al. *JAMA Network Open.*

eFigure. eConsultations and Referrals, and Primary Care Visits Over Time

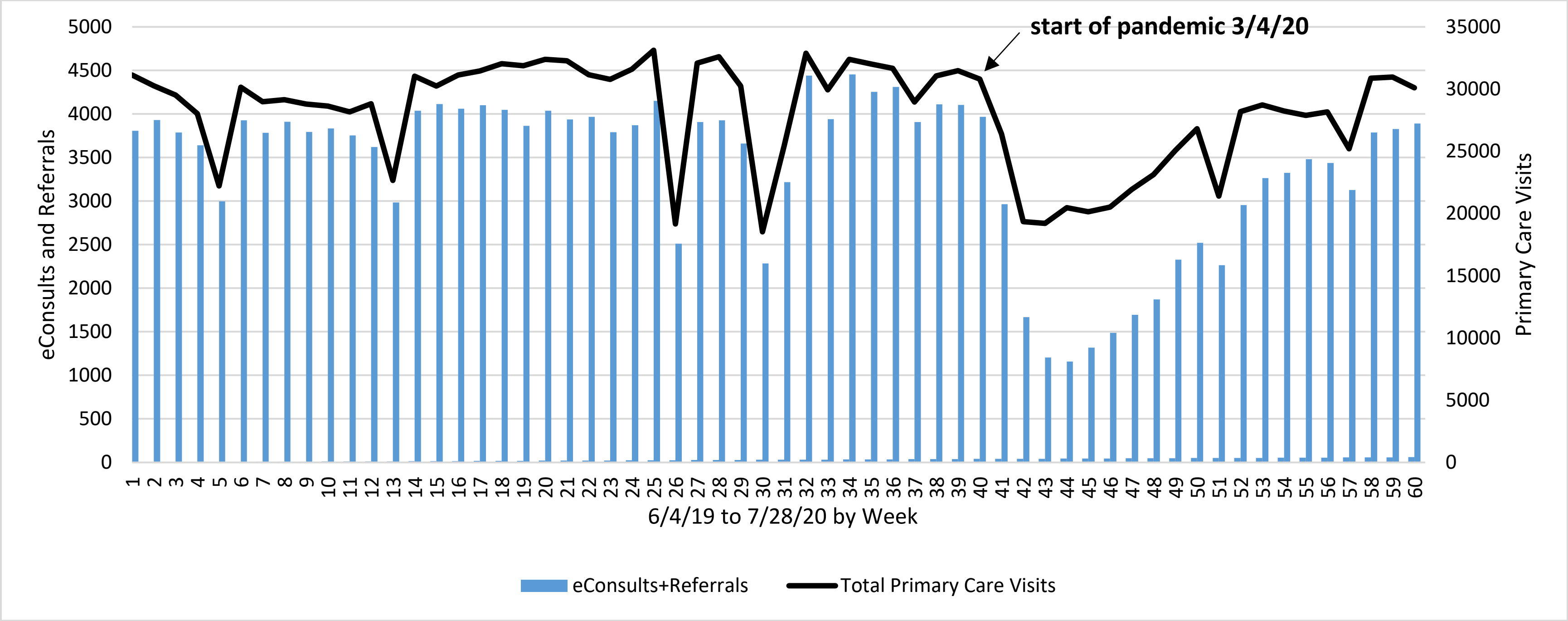

© 2022 Arora A et al. *JAMA Network Open.*

eTable 5. Number of eConsultations and Referrals per 1000 Primary Care Visits Before and During the Pandemic

	**Pre-pandemic (6/4/2019 – 3/3/2020)**			**During pandemic (3/4/2020 – 7/28/2020)**		
Academic Medical Center	Numerator (eConsults)	Denominator (primary care visits)	eConsults per 1,000 total primary care visits	Numerator (eConsults)	Denominator (primary care visits)	eConsults per 1,000 total primary care visits
A	1,421	175,375	8.1	818	72,505	11.3
B	1,828	255,448	7.2	868	104,724	8.3
C	2,139	212,247	10.1	1,084	84,540	12.8
D	1,740	79,292	21.9	845	46,386	18.2
E	2,317	353,714	6.6	1,098	194,218	5.7
F	259	78,083	3.3	128	31,287	4.1
Overall	9,704	1,154,159	8.4	4,841	533,660	9.1

	**Pre-pandemic (6/4/2019 – 3/3/2020)**			**During pandemic (3/4/2020 – 7/28/2020)**		
Academic Medical Center	Numerator (referrals)	Denominator (primary care visits)	Referrals per 1,000 total primary care visits	Numerator (referrals)	Denominator (primary care visits)	Referrals per 1,000 total primary care visits
A	15,685	175,375	89.4	6,745	72,505	93.0
B	39,021	255,448	152.8	15,413	104,724	147.2
C	30,826	212,247	145.2	10,119	84,540	119.7
D	25,564	79,292	322.4	8,744	46,386	188.5
E	25,554	353,714	72.2	8,935	194,218	46.0
F	2,427	78,083	31.1	743	31,287	23.7
Overall	139,077	1,154,159	120.5	50,699	533,660	95.0

© 2022 Arora A et al. *JAMA Network Open.*

eTable 6. Percentage of Telehealth Visits in Primary Care

	**Pre-pandemic (6/4/2019 – 3/3/2020)**			**During pandemic (3/4/2020 – 7/28/2020)**		
Academic Medical Center	Numerator (telehealth visits)	Denominator (primary care visits)	% Telehealth visits in primary care	Numerator (telehealth visits)	Denominator (primary care visits)	% Telehealth visits in primary care
A	975	175,375	0.6%	33,889	72,505	46.7%
B	0	255,448	0.0%	29,110	104,724	27.8%
C	75	212,247	0.04%	45,522	84,540	53.9%
D	1,064	79,292	1.3%	34,139	46,386	73.6%
E	20,21	353,714	0.6%	84,050	194,218	43.3%
F	117	78,083	0.2%	21,992	31,287	70.3%
Overall	4,252	1,154,159	0.4%	248,702	533,660	46.6%

© 2022 Arora A et al. *JAMA Network Open.*

eTable 7. Full Interrupted Time-Series Models

	Model 1: Overall eConsult Proportion	Model 2: eConsult Proportion (Non-English languages)	Model 2: eConsult Proportion (English only)	Model 3: eConsult Proportion (Payer type= Commercial)	Model 3: eConsult Proportion (Payer type= Medicaid)	Model 3: eConsult Proportion (Payer type= Medicare)	Model 3: eConsult Proportion (Payer type=Other)	Model 3: eConsult Proportion (Payer type= Uninsured or self-pay)
Pre-Pandemic Slope, %, (SE)	-0.00343 (-0.71)	0.0360 (1.52)	-0.00952 (-1.46)	-0.00902 (-1.32)	0.00920 (0.73)	0.00766 (1.53)	-0.0203 (-0.60)	-0.0652*** (-4.27)
First Week of COVID, Change in Intercept, %, (SE)	6.206*** (10.05)	8.481*** (6.35)	6.100*** (9.42)	6.938*** (9.99)	2.943*** (4.75)	5.870*** (8.84)	8.941*** (7.18)	-0.214 (-0.38)
During and after the 2nd Week of COVID-19 Pandemic, slope, %, (SE)	-0.29*** (-0.04)	-0.54*** (-0.09)	-0.28*** (-0.04)	-0.31*** (-0.05)	-0.15** (-0.04)	-0.33*** (-0.04)	-0.28** (-0.09)	0.02 (-0.04)
Change in Slope % , (SE)	-0.296*** (-6.41)	-0.576*** (-5.80)	-0.272*** (-5.68)	-0.306*** (-5.92)	-0.162** (-2.97)	-0.340*** (-7.10)	-0.265* (-2.41)	0.0866 (1.86)
Intercept %, (SE)	6.596*** (67.82)	8.348*** (15.82)	6.643*** (51.34)	6.945*** (50.41)	4.949*** (18.75)	5.923*** (55.62)	12.84*** (19.38)	6.083*** (17.71)
N	60	60	60	60	60	60	60	60
P-Value	* p<0.05, ** p<0.01, *** p<0.001"							

© 2022 Arora A et al. *JAMA Network Open.*
